# Memory Effect in the Spatial Series Based on Diamond and Graphite Crystals

**DOI:** 10.3390/molecules25225387

**Published:** 2020-11-18

**Authors:** Ludmila Grigoreva, Alexander Razdolsky, Vladimir Kazachenko, Nadezhda Strakhova, Veniamin Grigorev

**Affiliations:** 1 Faculty of Fundamental Physical and Chemical Engineering, Lomonosov Moscow State University, Leninskiye Gory 1/51, 119991 Moscow, Russia; ldg@physchem.msu.ru; 2 Department of Computer-Aided Molecular Design, Institute of Physiologically Active Compounds of the Russian Academy of Sciences, Severny proezd 1, 142432 Chernogolovka, Russia; rasd@ipac.ac.ru (A.R.); kazach@ipac.ac.ru (V.K.); strakh@ipac.ac.ru (N.S.)

**Keywords:** diamond, graphite, Hurst exponent, long memory

## Abstract

To study the relation between the structure of a compound and its properties is one of the fundamental trends in chemistry and materials science. A classic example is the well-known influence of the structures of diamond and graphite on their physicochemical properties, in particular, hardness. However, some other properties of these allotropic modifications of carbon, e.g., fractal properties, are poorly understood. In this work, the spatial series (interatomic distance histograms) calculated using the crystal structures of diamond and graphite are investigated. Hurst exponents H are estimated using detrended fluctuation analysis and power spectral density. The values of H are found to be 0.27–0.32 and 0.37–0.42 for diamond and graphite, respectively. The calculated data suggest that the spatial series have long memory with a negative correlation between the terms of the series; that is, they are antipersistent.

## 1. Introduction

One of the areas in modern natural science, which attracts the attention of researchers, is fractal geometry and nonlinear dynamics [1,2,3]. Here, the objects of inquiry can be time or spatial series formed during experimental observations or theoretical calculations. This approach is applied in areas such as astronomy (sun spots) [4], seismology (Earth crust vibrations) [5], physiology (electroencephalograms) [6], meteorology (weather observation data) [7], econophysics (stock quotation) [8], bioinformatics (protein dynamics) [9], and chemistry (concentration changes) [10]. The following two basic problems are solved here: the classification of series and the prediction of their behavior.

To describe dynamic systems quantitatively, researchers have used various quantities to characterize their complexity, ordering, and other properties. One of them is Hurst exponent H, which changes in the range from 0 to 1 and can be used to classify time and spatial series into random and nonrandom series in order to identify a long-range dependence (long memory). In this case, memory is considered in the sense that the future of the process is predicted as a function of its current state. Quantity H is closely related to fractal geometry, which studies the objects characterized by self-similarity [11]. Here, a scaling region is unlimited only in the case of mathematical objects; in other cases, it is local. Fractal signals, which include time (space) series, are often called long memory processes [12]. They can be classified according to H into three groups. H > 0.5 corresponds to a process with a long-range dependence and a positive correlation. If H < 0.5, the process has a long-range dependence with a negative correlation. At H = 0.5, we observe either a random process or a process with a short-range dependence [13].

Hurst exponents are calculated by various methods, such as the rescaled range (R/S) method [14], detrended fluctuation analysis (DFA) [15], power spectral density (PSD) [16], and fractal wavelet analysis [17]. These methods are based on estimating the exponent of a power function, in which a certain statistic is a dependent variable and a scale (time or spatial) variable is an independent variable. The exponent is fractional in the case of fractal objects. Here, the R/S method is a direct technique for estimating H and other approaches need recalculation.

A new approach to studying the relation between the structure of a chemical compound and its properties is the use of an interatomic distance histogram as a spatial series [18,19,20]. In particular, when the homologous series of organic compounds were studied, the series length was found not to affect the Hurst exponent. A simple regression model, which relates H to the maximum valence bond length, was proposed to explain the differences between homologs.

A classic example of the influence of the structure of a molecule on its properties is represented by the allotropic modifications of carbon, namely diamond and graphite [21]. This group of substances is widely used in practice and has been widely studied. For example, diamond is characterized by an extremely high hardness, which is mainly caused by its crystal structure. Carbon atoms in diamond are in the state of sp^3^ hybridization. Each atom is located at the center of a tetrahedron and is connected to four other atoms by a high-strength covalent bond. Tetrahedra form a three-dimensional network consisting of layers of six-membered rings. Another modification of carbon is graphite, which has a low hardness. It has a layered structure consisting of six-membered rings, in which every sp^2^-hybridized carbon atom is covalently bonded to three other atoms. Although the physicochemical and biological properties of diamond and graphite have been widely studied, their fractal properties are poorly understood and need further investigation.

The purpose of this work is to calculate and analyze the Hurst exponents of the spatial series based on the interatomic distance histograms of diamond and graphite crystals using DFA and PSD.

## 2. Results and Discussion

The calculated results are shown in Table 1. The data obtained by the two applied methods are seen to agree with each other. The range of changing α(H_α_) is 0.27 ÷ 0.40; that of −β is 0.17 ÷ 0.38; and H_β_, 0.31 ÷ 0.42. Diamond crystals have lower values of H_α_ and H_β_ as compared to graphite crystals. The randomization data (H_α_^rand^, H_β_^rand^) correspond to the theoretical expectation (H = 0.5), which confirms the reliability of the calculated results.

In terms of the dichotomic model [12], time and spatial series can be attributed to stationary fractional Gaussian noise (fGn) (α < 1, β < 1) or nonstationary fractional Brownian motion (fBm) (α > 1, β > 1), depending on α and β. These processes are interrelated: integration transforms fGn into fBm and differentiation transforms fBm into fGn. Since all calculated values fall in the ranges α < 1 and β < 1, all interatomic distance histograms of the crystals can be attributed to the fGn process.

The Hurst exponent is known to be a quantitative measure of memory for time and spatial series [13,22,23]. At H = 0.5, a series is random and the previous, current, and subsequent values are not related. At 0.5 < H < 1, data exhibit a long-range dependence, which is characterized by a positive correlation between the terms of series. An increase in the dependent variable at present is most likely to be retained in the future, and, vice versa, a current decrease is most likely to continue in the future. Such series are called persistent. The range 0 < H < 0.5 is characteristic of long memory with a negative correlation between the terms of series, which is inherent in antipersistent systems. They are characterized by a rapid increase/decrease in the dependent variable in response to a decrease/increase, which leads to oscillations about certain means. As follows from the aforesaid, all spatial series under study are antipersistent (see Table 1).

**Figure 1:**
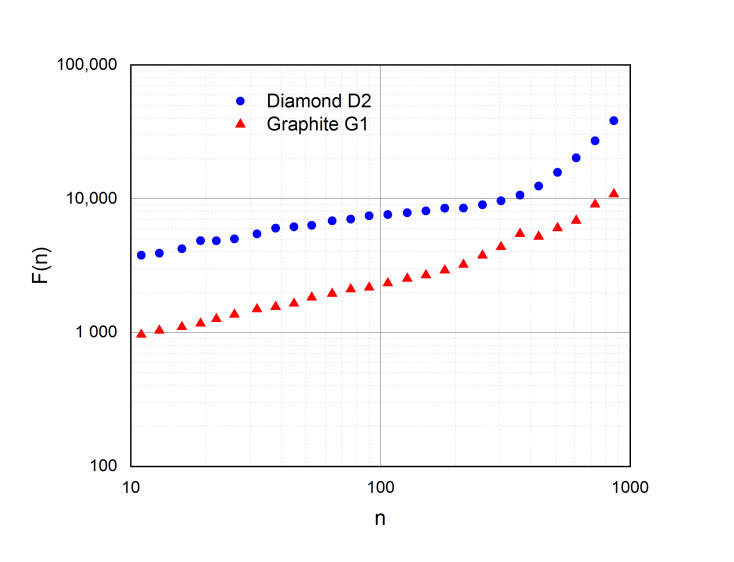
Scale invariance regions of diamond and graphite according to DFA data.

The interatomic distance histograms under study are statistical fractals characterized by a limited scaling region. In particular, according to DFA data, the scale invariance region of diamond crystals is from n_min_ = 11 to n_max_ = 304 (Figure 1), which corresponds to distances r_min_ = 0.11 (11 × 0.01) Å and r_max_ = 3.04 (304 × 0.01) Å. In the case of graphite crystals, we have n_min_ = 11 (r_min_ = 0.11 Å) и n_max_ = 215 (r_max_ = 2.15 Å). At the end of these ranges, an inflection point forms and the angle between log(F(n)) and log(n) increases. However, the new linear segments are short in the scale region and have a small number of points; therefore, it is difficult to estimate their fractal parameters.

Note that the position of the inflection point in the scaling curve of diamond crystals (3.04 ÷ 3.62 Å) approximately corresponds to the lattice parameter (3.56 ÷ 3.567 Å) (Table 2). The inflection point characteristics of graphite crystals (2.15 ÷ 2.56 Å) agree well with the translation vector a and b lengths ~2.5 Å. An increase in the values of H in the diamond-graphite row (0.27 < 0.40) is accompanied by a decrease in the average position of the inflection point (3.33 > 2.36 Å) and a shortening of the scale invariance region.

One of the fundamental problems in chemistry is the structure–property relationship. In the applied research devoted to this problem, the approach based on the methodology of quantitative structure–activity/property relationship (QSAR/QSPR) is widely used. One of the elements of this approach is to describe the structure of chemical compounds as a matrix of various physicochemical, topological, electronic, and other quantitative characteristics (descriptors). Taking into account the complexity of a molecular structure, we can state that the search for new descriptors with certain information is a challenging problem [24]. Note that, in QSAR/QSPR studies, some descriptors are used to describe the “structure” of chemical compounds (independent variables) and others act as “properties” (dependent variable). Earlier, we proposed a number of descriptors to describe new fractal properties of molecules. They were initially applied to molecules in a gas phase. In addition, the influence of structural changes on the properties and relationship of fractal descriptors with other well-known descriptors was investigated. In particular, in [19,20], we calculated and studied the behavior of the Hurst exponents of some organic molecules in a gas phase and found that the series formed using interatomic distance histograms had H > 0.5; that is, they are persistent. However, as was found in this work, the spatial series of the two crystalline allotropic modifications of carbon are antipersistent. The resulting discrepancy can be associated with different phase states of the compounds under study (gas or crystal). Further investigations are required to reveal the cause of this discrepancy.

## 3. Materials and Methods

We studied the geometric structures of diamond and graphite crystals. The three-dimensional structures of the substances were constructed with the HyperChem software package [25]. For calculations, we used *.cif files taken from various sources. The lattice parameters and the atomic coordinates in the unit cells were determined using the CrystalMaker software package [26]. The crystal sizes were 343 (7 × 7 × 7) unit cells (2744 atoms) for diamond (D1, D2) and 512 unit cells (8 × 8 × 8) (2048 atoms) for graphite (G1, G2). Table 2 gives the crystallographic parameters of diamond (cubic system, space group Fd3m) and graphite (hexagonal system, space group P6_3_/mmc).

**Figure 2:**
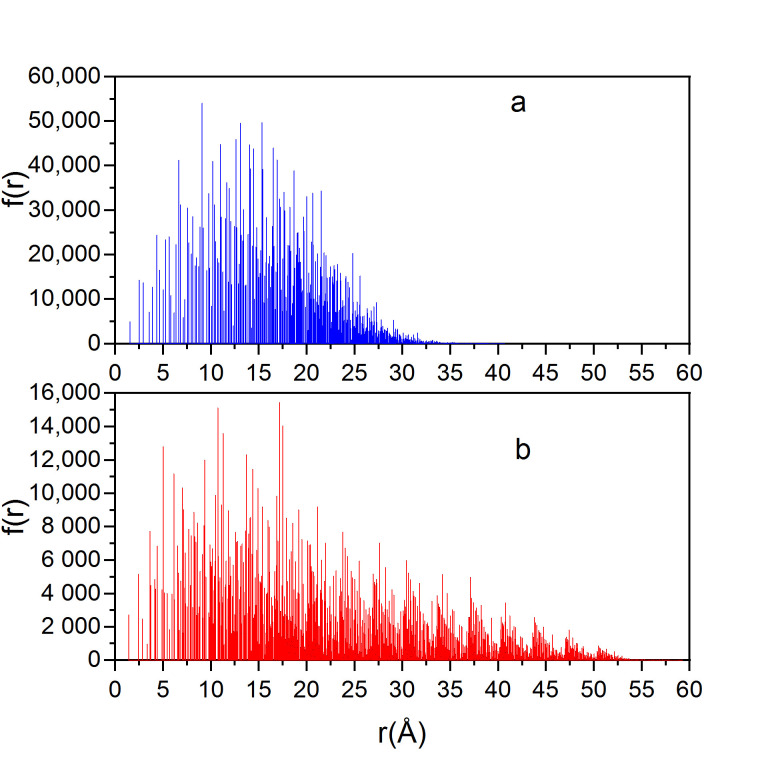
Interatomic distance histograms of (**a**) diamond D2 and (**b**) graphite G1.

The three-dimensional structures of the crystals were converted into interatomic distance histograms at a step of 0.01 Å (Figure 2). The y-axis is the frequency, i.e., the number of cases in each interval. The obtained spatial series were studied by DFA and PSD.

**Figure 3:**
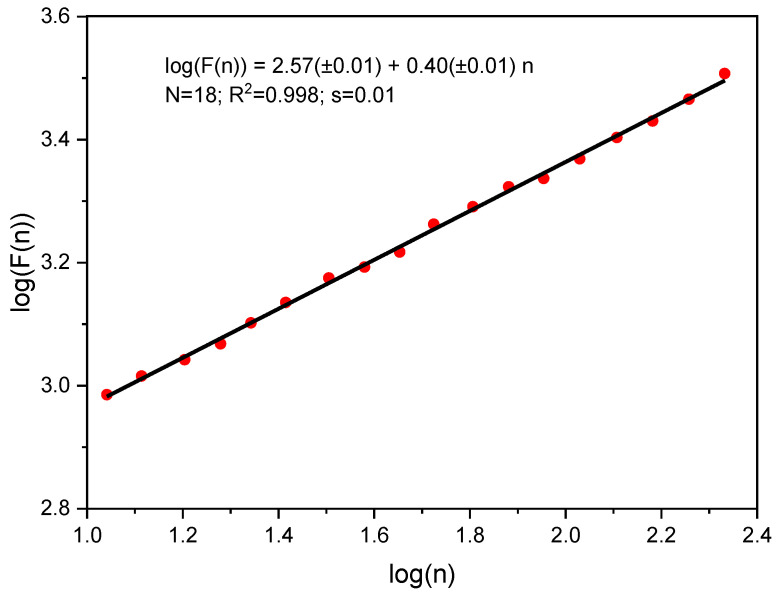
Function F(n) vs. block size n for graphite G1.

The DFA algorithm [15,29] can be represented as the following sequential steps: (1) discrete X series containing M readings was transformed by subtracting the mean (µ) and summation y_k_ = Σ^k^_i=1_(X_i_ − µ), (2) the obtained series was divided into nonoverlapping blocks of length n, (3) local trend y_k,n_ was estimated in each block using the least squares method, (4) fluctuation function F(n) = ((Σ^M^_k=1_(y_k_ – y_k,n_)^2^)/M)^0.5^ was determined for each block, and (5) exponent α was calculated by double logarithmic transformation of the equation F(n) = const n^α^. In this work, we used a linear function to estimate a local trend. The minimum and maximum block sizes were 10 and M/4, respectively. Figure 3 shows an example of the calculation.

**Figure 4:**
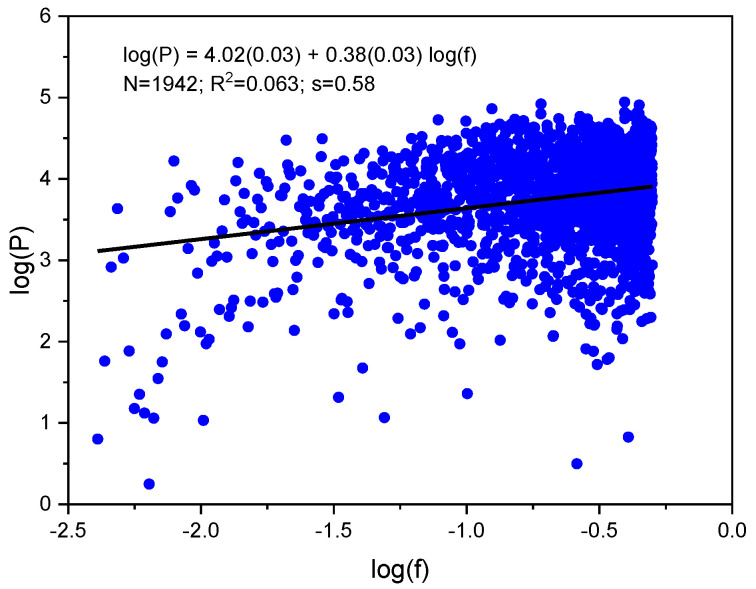
Power spectrum of diamond D2.

To perform PSD analysis, we used the fast Fourier transform (FFTRF) from the IMSL (International Mathematics and Statistics Library) 7.0 [30]. An initial interatomic distance histogram was converted into the frequency (f) dependence of power density (P). This dependence was subjected to double logarithmic transformation to determine spectral exponent β. Figure 4 shows an example of the calculation.

Using the dichotomic model [12] and parameters α and β, we calculated the Hurst exponents with the formulas H_α_ = α (α < 1), H_α_ = α − 1 (α > 1), H_β_ = (β + 1)/2 (β < 1), and H_β_ = (β − 1)/2 (β > 1).

The statistical parameters of regression equations were as follows: N was the number of points, R^2^ was the squared coefficient of linear correlation, and s was the standard deviation.

The Hurst exponents were subjected to internal validation by calculating H^rand^ for the randomized series of the initial data (interatomic distance histograms). In this case, the values of f(r) did not change but the order of their appearance in a spatial series changed. To this end, we used 10 random sets of data and averaged the results obtained.

## 4. Conclusions

Using available crystallographic data, we constructed ideal crystal structures of diamond and graphite. Three-dimensional atomic coordinates were used to calculate interatomic distance histograms. The obtained spatial series were analyzed by DFA and PSD. When analyzing Hurst exponents, we found that the series under study had H < 0.5; that is, they are antipersistent. When studying scale invariance regions, we revealed inflection points, the position of which correlates with the lattice parameters. The data obtained can be used for the computer-aided molecular design of new substances and materials with allowance for their fractal properties.

## Figures and Tables

**Table 1:** Parameters (α, β), Hurst exponents (H), and standard errors (Δ), calculated by detrended fluctuation analysis (DFA) (α, H_α_, H_α_^rand^) и power spectral density (PSD) (β, H_β_, H_β_^rand^).

Crystal	α ± Δ	H_α_ ± Δ	H_α_^rand^ ± Δ	−β ± Δ	H_β_ ± Δ	H_β_^rand^ ± Δ
D1	0.27 ± 0.01	0.27 ± 0.01	0.50 ± 0.02	0.37 ± 0.03	0.32 ± 0.02	0.51 ± 0.02
D2	0.27 ± 0.01	0.27 ± 0.01	0.49 ± 0.02	0.38 ± 0.03	0.31 ± 0.02	0.50 ± 0.01
G1	0.40 ± 0.01	0.40 ± 0.01	0.49 ± 0.02	0.26 ± 0.03	0.37 ± 0.01	0.50 ± 0.01
G2	0.39 ± 0.01	0.39 ± 0.01	0.51 ± 0.02	0.17 ± 0.03	0.42 ± 0.01	0.51 ± 0.01

**Table 2:** Unit cell parameters of diamond and graphite.

Crystal	a	b	c	α	β	γ	Z	Source
D1	3.56	3.56	3.56	90	90	90	8	[25]
D2	3.567	3.567	3.567	90	90	90	8	[27]
G1	2.464	2.464	6.711	90	90	120	4	[27]
G2	2.511	2.511	6.72	90	90	120	4	[28]
